# Potential Biotechnological Applications of Venoms from the *Viperidae* Family in Central America for Thrombosis

**DOI:** 10.3390/toxins16030142

**Published:** 2024-03-08

**Authors:** Jorge Eduardo Chang Estrada, Taissa Nunes Guerrero, Daniel Fernando Reyes-Enríquez, Erica Santos Nardy, Roseane Guimarães Ferreira, Cristian José Ruiz Calderón, Irmgardt A. Wellmann, Kaio Murilo Monteiro Espíndola, Alejandro Ferraz do Prado, Andreimar Martins Soares, Marcos Roberto de Mattos Fontes, Marta Chagas Monteiro, Russolina Benedeta Zingali

**Affiliations:** 1 Instituto de Bioquímica Médica Leopoldo de Meis CCS, Universidade Federal do Rio de Janeiro, Rio de Janeiro 21941-902, RJ, Brazil; jorge.estrada@bioqmed.ufrj.br (J.E.C.E.); taissa.guerrero@bioqmed.ufrj.br (T.N.G.); daniel.enriquez@bioqmed.ufrj.br (D.F.R.-E.);; 2 Postgraduate Program in Neuroscience and Cell Biology, Federal University of Pará, Belém 66075-110, PA, Brazil; roseguiferreira@yahoo.com.br (R.G.F.); martachagas@ufpa.br (M.C.M.); 3 Department of Biochemistry and Microbiology, Universidad del Valle de Guatemala, Guatemala City 01015, Guatemala; rui14294@uvg.edu.gt; 4 Postgraduate Program in Tropical Medicine, State University of Amazonas, Manaus 69005-010, AM, Brazil; irmgardtina@gmail.com; 5 Faculty of Medical Sciences, Universidad de San Carlos de Guatemala, Guatemala City 01015, Guatemala; 6 Postgraduate Program in Pharmaceutical Sciences, Faculty of Pharmacy, Federal University of Pará, Belém 66075-110, PA, Brazil; kaioespindola@hotmail.com; 7 Laboratory of Pharmacology and Toxicology of Cardiovascular System, Institute of Biological Sciences, Federal University of Pará, Belém 66075-110, PA, Brazil; alejandrofp@ufpa.br; 8 Laboratory of Biotechnology and Education Applied to One Health (LABIOPROT), Oswaldo Cruz Foundation, FIOCRUZ, RONDÔNIA, Federal University of Rondônia, UNIR, Porto Velho 76812-245, RO, Brazil; andreimar.soares@fiocruz.br; 9 Sao Lucas University Center, SÃO LUCAS PVH, Porto Velho 76804-414, RO, Brazil; 10 Western Amazon Research and Knowledge Network of Excellence (RED-CONEXAO), Basic and Applied Toxinology Research Network (RED-TOX), the National Institute of Science and Technology of Epidemiology of the Western Amazon (INCT EpiAmO), Porto Velho 76812-245, Ro, Brazil; marcos.fontes@unesp.br; 11 Institute for Advanced Studies of the Sea (IEAMar), Universidade Estadual Paulista (UNESP), São Vicente 11350-011, SP, Brazil; 12 Department of Biophysics and Pharmacology, Institute of Biosciences, Universidade Estadual Paulista (UNESP), Botucatu 18618-970, SP, Brazil

**Keywords:** *Viperidae*, venom, envenomation, biomolecules, thrombosis

## Abstract

Central America is home to one of the most abundant herpetofauna in the Americas, occupying only 7% of the continent’s total area. Vipers and lizards are among the most relevant venomous animals in medical practice due to the consequences of envenomation from the bite of these animals. A great diversity of biomolecules with immense therapeutic and biotechnological value is contained in their venom. This paper describes the prominent leading representatives of the family *Viperidae*, emphasizing their morphology, distribution, habitat, feeding, and venom composition, as well as the biotechnological application of some isolated components from the venom of the animals from these families, focusing on molecules with potential anti-thrombotic action. We present the leading protein families that interfere with blood clotting, platelet activity, or the endothelium pro-thrombotic profile. In conclusion, Central America is an endemic region of venomous animals that can provide many molecules for biotechnological applications.

## 1. Introduction

In a geopolitical sense, Central America comprises seven countries: Belize, Guatemala, El Salvador, Honduras, Nicaragua, Costa Rica, and Panama. These countries form an extension of 523,780 km^2^ combined, which also encompasses the Mesoamerican biodiversity hotspot that extends from the north of Guatemala to the south of Panama [1]. In a biogeographic sense, this extension of land is regarded as a combination of the Mexican transition zone and Neotropical region—this last one also containing the Caribbean subregion and Mesoamerican dominion. The region’s diversity is partly due to the highly changing provinces in terms of altitude, humidity, temperature, and energy availability in an otherwise small region. This area is divided into seven provinces [1], producing highly competitive niches that promote variation and speciation in fauna and flora.

**Figure 1:**
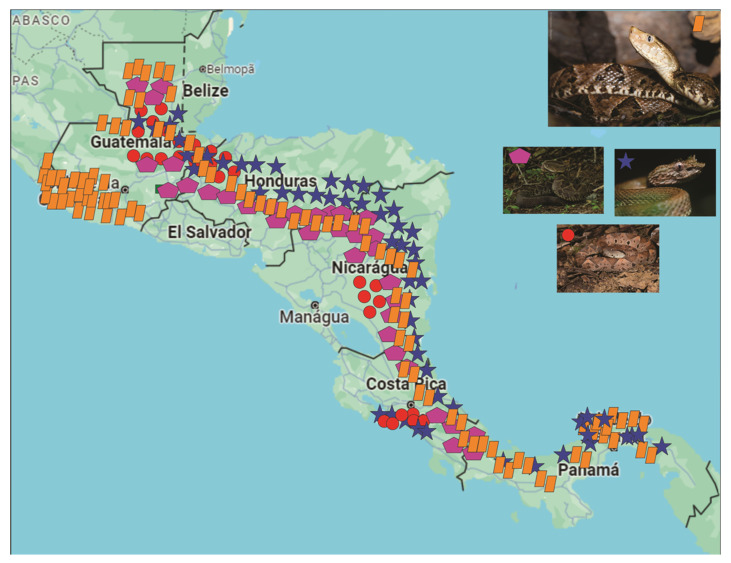
Distribution of some species of the *Viperidae* family that present potential biotechnological compounds against thrombosis and other diseases. These snakes are responsible for the most ophidic accident cases in the Central American region. Red, *Metlapilcoatlus* sp. and *Atropoides* sp.; violet, *Crotalus simus*; blue, *Bothriechis* sp.; and orange, *Bothrops asper*. Photos by Andres Novales and Francisco Obregón. The map was adapted from Google Maps. The photos of the representative snakes for each genus are demonstrative since species have many morphological variations.

Central America has an incredible variability of venomous reptiles, grouped into 33 species of *Viperidae*, 19 species of *Elapidae*, and two species of *Helodermatidae* [2,3]. One of the most important snake families in this group, *Viperidae*, is composed of species of the genera *Bothrops*, *Agkistrodon*, *Atropoides*, *Bothriechis*, *Crotalus*, *Cerrophidion*, *Lachesis*, *Metlapilcoatlus*, and *Porthidium* (Figure 1), with their principal venom components, snake venom metalloproteinases (SVMPs), phospholipases A_2_ (PLA_2_s), snake venom serine proteases (SVSPs), and L-amino-acid oxidases (LAAOs) in Table 1.

However, despite the envenoming bite of these animals, the components of the *Viperidae* venoms offer alternative avenues for designing new drugs in medical therapy [4,5,6]. For example, we have *Crotalus* snake venom components with anti-tumor, anti-inflammatory (crotoxin), and anti-cancer (tzabcanin) activities [4,5,6,7,8] that can be further explored.

This review aims to describe the most studied species of the *Viperidae* family of the Central American region, emphasizing the potential biotechnological uses of their venom components against thrombosis and showing the need for studies from the Central American species. To demonstrate the biotechnological potential of these venoms, we focus on molecules already purified from other *Viperidae* venoms that act on the hemostatic system and can be used to develop new anti-thrombotic drugs, such as batroxobin from *Bothrops atrox moojeni*, crotoxin and L-amino oxidases from *Crotalus durrisus terrificus*, and others described below [9,10,11].

**Table 1:** Relative abundance of protein families in the venom from Central America’s main genera and species.

Genera	Described Species	Principal Protein Families Percentages (%)				Reference
		SVMPs	PLA_2_s	SVSPs	LAAOs	
*Agkistrodon*	*A. bilineatus*	21–33	32–42	9–23	0.8–9	[12,13,14,15,16]
*Atropoides*	*A. picadoi*	66	9.5	13.5	2.2	[17,18,19,20]
*Bothriechis*	*B. schlegelii*	17.7	43.8	5.8	8.9	[21,22,23]
*Bothrops*	*B. asper*	41	29	18	9	[24,25]
*Cerrophidion*	*C. godmani*	32.8	23.4	19.1	<10	[26,27]
*Crotalus*	*C. simus*	28	22	30	17	[10]
*Lachesis*	*L. melanocephala*	49.2	13.4	21	3.6	[28,29]
*Metlapilcoatlus*	*M. mexicanus*	18.2	36.5	22	9.1	[20,30]
*Porthidium*	*P. nasutum*	52.1	11.6	9.6	9.8	[31]

Note. The references of the venom composition have previous taxonomic names of the snake’s species.

## 2. *Viperidae* Family

The *Viperidae* family, also called vipers, comprises approximately 260 species of venomous snakes grouped into four subfamilies: *Azemiopinae*, *Causinae*, *Crotalinae*, and *Viperinae* [17,32]. These snakes are characterized by possessing a mechanically more sophisticated system of venom injection, composed of a pair of secretory glands that produce a complex of enzymes and proteins that aim to paralyze and kill their prey [17,33]. The venom has immediate action, functioning at the moment of the bite as a hemotoxic pre-digestive that initiates a process of tissue destruction, helping the animal when feeding [4]. For this reason, the *Viperidae* family is considered a group of great medical importance because its bite can lead a person to death [34,35].

The clinical aspect of the envenomation by these vipers is characterized by a complex series of rapid onset local effects that develop in the vicinity of the site where the venom was inoculated, such as pain, edema, necrosis, local bleeding, bruising, and blistering, followed by effects such as nausea, vomiting, fever, systemic bleeding, hypotension, oliguria, or anuria [36,37,38]. When treatment is not initiated promptly, the local effects can result in permanent sequelae to tissue damage, with lasting consequences [36,37,38,39]. Venom distribution in moderate and severe cases produces systemic manifestations such as hemorrhage, coagulopathy (systemic bleeding), and, in some cases, acute renal failure (oliguria or anuria), neurotoxicity, and cardiovascular shock (hypotension) [36,37,38,39].

### 2.1. Agkistrodon

*Agkistrodon* is a genus of vipers anatomically characterized by a vertical elliptical pupil and a large venom gland in the temporal region with a canalicular fang in the mandible region, followed by a series of smaller fangs [12,13]. The skull is relatively large and has short tusks [12,13]. The tail can be of moderate to long size, and newborns and juveniles of all species of this genus have a colored tail tip (yellow, white, or pink), turning to faded green, gray, and black when these animals become adults [12,13]. These snakes usually attract their prey with their tails, simulating movements resembling waving (called caudal attraction) [13]. The *Agkistrodon* genus is distributed throughout the northwest, central, and coast of the United States, Mexico, and mainly in Central America, whose main species is *A. bilineatus* [15].

#### *Agkistrodon bilineatus* 

This species, also known as cantils, is found at low and moderate elevations along the Pacific coast to the southern foothills of Sonora, Mexico; Guatemala; Honduras; and northwestern Costa Rica [13,14,15] *A. bilineatus* is a heavy animal with a large head and a long tail [12,13]. The adult can be 80 cm in total length (TL) (with a record of 138 cm). The length of the head is about 17–27% of TL in males and approximately 14–25% of TL in females [11,12]. The snake has some conspicuous pale stripes on each side of the head, except for adults from southern Honduras, Nicaragua, and Costa Rica [12,13,14]. These stripes extend from the rostrum throughout the body [12,13]. The coloration of this adult species is dark gray to almost black, brown, and dark gray–brown, depending on geographic variation. Neonates and juveniles are brown with crossbands and white-spotted side edges, and these spots gradually fade with maturity. The tails of these young animals are bright yellow to attract their prey [12,13,14]. *A. bilineatus* feeds on small mammals, birds, reptiles, amphibians, and fish. In addition, it has a nocturnal habit and is terrestrial to semi-aquatic (found near water bodies) [14]. This viper has an aggressive, feared temperament and a very active venom that can kill its prey within hours [40,41].

The venom of this species is well known for its hemotoxic action, composed of 31.5–42% phospholipase A_2_ (PLA_2_), 21.0–33.1% snake venom metalloproteinases (SVMPs), and 8.9–22.5% snake venom serine proteases (SVSPs). The remaining venom components are L-amino-acid oxidases (LAAO) and some non-enzymatic proteins. *A. bilineatus* venom (currently divided into *A. bilineatus* and *A. russeolus*) is reported to be highly vasculotoxic and ten times more hemorrhagic and edematizing than *Bothrops asper* venom [16,42].

A few molecules interfering in the hemostatic system were described in this venom. Bilineobin is a thrombin-like enzyme with low activity and forms fibrin, generating fibrinopeptides A and B; its sequence is similar to batroxobin [43,44]. A protein C activator was also described. It is a serine protease with high homology with Protac, which was used to test Factor V resistance and protein C pathway defects [45,46].

### 2.2. Atropoides 

Vipers of the genus *Atropoides* are called jumping pit vipers, as they can jump over their prey during a potential attack [17,18,19,47]. These vipers are characterized morphologically by their thick, robust, and short-length bodies [19,47]. The head is large, the eyes are relatively small, the snout is round, and the tail is short, representing about 15% of the body length [19,47]. The color of these snakes ranges from gray, brown, or reddish, covered with various lateral and dorsal spots, and their distribution includes Mexico and broader Central America [17,18,19,47]. The most representative species of this genus is *A. picadoi* [19,47].

The species of the genus *Atropoides* are close relatives of the genus *Metlapilcoatlus*. But their venoms are diverse [18], with a higher predominance of Zinc-dependent metalloproteinases (SVMPs) in the genus *Atropoides* and a higher predominance of phospholipases A_2_ (PLA_2_s) in the venom of *Metlapilcoatlus*. Despite the importance of the genus in Costa Rica, characterization of the venom compounds still needs to be accomplished, and few studies have been focused on the venom’s coagulotoxic effect, regardless of the venom’s anti-thrombotic potential [48].

#### *Atropoides picadoi* 

This species is distributed in low and moderate elevations in Costa Rica, western Panamá, and Atlantic slopes, inhabiting subtropical-humid and mountainous-humid rainforests [20]. Anatomically, the snake has a highly thick body, reaching about 75–95 cm TL, and can exceed one meter in length [19]. The top of the head is dark brown or reddish brown, with or without irregular dark brown markings [19]. A broad dark brown color follows the entire length of its body to the jaw. A vague to moderately distinct dark spot is present below the eye; sometimes, another is below the lateral well. They have about 25 to 30 dorsal patches zigzagging throughout the body. The belly is white to yellow, with an irregular brown pattern [19].

The venom of *A. picadoi* predominantly comprises SVMPs (66.4%) [18]. To our knowledge, no protein that affects hemostasis has been purified or characterized in this venom.

### 2.3. Bothriechis

The name *Bothriechis* is derived from the Greek *bothos*, which means pit, and *echis*, which represents viper, due to the prominent heat-sensitive pit between the nostril and the eyes [22]. This pit acts as an infrared receiver, allowing the snake to accurately detect and strike warm-blooded prey, even in complete darkness [22]. These vipers are also called palm pit vipers and are distributed throughout Central and South America [49,50,51] in humid and mountainous isolated forests. They have nocturnal habits, are arboreal [22,49,50,51], and feed on small rodents, frogs, lizards, small birds, and bats [22,23,51]. The body of the genus *Bothriechis* is slim, medium, and robust, with a short, prehensile tail that represents 15% of body length; usually, an adult species can reach 70 and 85 cm in length [3]. The snout is flat and round, and the color of these snakes is greenish [49]. We will focus on some of this genus’s main species: *Bothriechis aurifer*, *B. bicolor*, *B. thalassinus*, and *B. schlegelii* [41]. Also, other members, such as *B. nigroviridis*, *B. supraciliaris*, *B. rowleyi*, *B. marchi*, and *B. lateralis*, demonstrated biotechnological potential that this review will not describe [52].

Recently, the crude venoms of *B. aurifer*, *B. schlegelii*, and other species were analyzed for their ability to interfere with blood clotting. *B. schlegelii* showed a significant activity inducing fibrinogen clotting, although forming weak clots, with a pseudo-coagulant effect [52].

#### 2.3.1. *Bothriechis aurifer*

*B. aurifer*, known as palm pit viper and spotted yellow, can be found at an altitude of 1200–2300 m in cloudy forests in the eastern mountains of Chiapas (Mexico) and northwestern and central Guatemala [3]. This species has a dorsal pattern of yellow spots with a black border diffused between adjacent spots and dorsal scale lines in the middle of the body [3]. The venom of this snake is composed of SVMPs (33 to 39% of the venom proteome), lectin type C proteins (CTLs; 11 to 16%), bradykinin-potentiating peptide (BPP)-like molecules (10 to 13%), and cysteine-rich secretory proteins (CRISPs; 5–10%) [22]. No description of isolated components has been made so far.

#### 2.3.2. *Bothriechis bicolor*

*B. bicolor* (Guatemalan palm pit viper) is characterized by a uniform green and black postocular stripe on the head. Its interstitial skin and borders of some scales are often blue. There are 8–11 intersupraoculars, 8–12 supralabials, 9–14 infralabials, 21 midbody scale rows, 157–175 ventrals in males, and 57–65 ventrals in females. The TL is generally 60–70 cm. This snake inhabits eastern and central southern Guatemala, southeastern Mexico, and Honduras. This viper prefers rain and cloudy forests between 500 and 2000 m in altitude [22]. The venom components of this species are mainly D49-PLA_2_s [3]. The venom of this species has been poorly explored.

#### 2.3.3. *Bothriechis thalassinus*

This species, also known as the Merendón palm pit viper, is characterized by being arboreal and having a prehensile tail and a combination of scales as follows: 5–9 keel intersupraocular, 23–25 keel interictal, 161–168 ventral, and 60–67 subcaudal, as well as usually 21 dorsal scale lines in the middle of the body [3]. The TL of *B. thalassinus* ranges from 40 cm to just over 80 cm, showing the back of the body and the green leaf-like part of the head changing to a yellowish green in the lateral segments, including ventral and subcaudal parts, which are usually turquoise with spots on the head and back [3]. This snake inhabits the Merendón Mountain Range, shared between Guatemala and Honduras, Cerro Santa Bárbara and nearby forests in Honduras, Cerro del Mono and Sierra Caral in Guatemala, and also in the highlands between the borders of El Salvador, Honduras, and Guatemala [53,54].

*B. thalassinus* venom consists of about 33–39% of SVMPs, 11–16% of CTLs, 10–13% of BPPs, and 5–10% of CRISPs. One remarkable fact is this venom’s lack of PLA_2_ proteins [3,22]. No protein acting on hemostasis was purified from this venom.

#### 2.3.4. *Bothriechis schlegelii*

*B. schlegelii* (Eyelash viper) is a viper of nocturnal and crepuscular activity with arboreal and terrestrial habits [55]. This animal feeds on frogs, birds, rodents, bats, and skunks and can be found in rainforests, subtropical and tropical forests, and urban and rural areas [3,21,22]. Its location includes Central America from southern Mexico, passing to Belize, Guatemala, Honduras, Nicaragua, Costa Rica, Panama, and South America (Colombia, Ecuador, and Peru) [21,22]. Morphologically, this species has a short, blunt, prehensile tail and undivided sub-flows, with small supraciliary scales in the form of spines between the ocular and supraocular regions (cilia or horns). Its head and eyes are large, with elliptical vertical pupils, and muzzle slightly pointed at the dorsal view, of minor to moderate size. Adult females are robust and larger than males (35–82 cm and 38–69 cm, respectively) [23]. The individuals of *B. schlegelii* have a coloration that can vary between green, yellow, and deep red, depending on the region [3,21,22].

The prominent toxin families of this species’ venom are SVMPs (55%) and myotoxic PLA_2_s (44%), respectively [56]. Angulo and Lomonte (2005) purified one PLA_2_ with myotoxic and mild anti-coagulant activity [56].

### 2.4. Bothrops

*Bothrops*, also known as lancehead pit vipers, are found in tropical Latin America, from northeastern Mexico to Argentina, and the southern part of the Caribbean islands [3,57]. Snakes of this genus are terrestrial or semi-arboreal, nocturnal, and prefer rainy periods for breeding [3]. When young, these animals feed on exothermic prey, and when they become adults, they change to endothermic prey (rodents, birds, marsupials, and larger animals) [24]. The *Bothrops* are responsible for most snakebite accidents because they are large animals (can reach up to 200 cm in total length) and are enigmatic in color, especially *Bothrops asper* [57].

*Bothrops* crude venoms exhibit diverse effects on hemostasis, giving rise to various clinical outcomes, ranging from local bleeding to thrombosis and/or systemic bleeding. The underlying cause of bleeding has been definitively attributed to direct multifocal toxicity affecting vessels, platelets, and coagulation rather than disseminated intravascular coagulopathy [58]. While microthrombosis in *Bothrops* envenomation is infrequently documented compared to bleeding, the initial fibrin deposition resulting from venom-induced hemostasis disorders appears to lack immediate clinical consequences [59]. However, the increased occurrence of complications, such as acute kidney injury (AKI), suggests a broader impact of hemostasis disorders than initially understood [60].

#### *Bothrops asper* 

This species (called *Fer-de-lance* or *terciopelo*) inhabits regions of the Caribbean lowlands, the Atlantic coast of Tamaulipas (Mexico), the Yucatan peninsula, Central America, northeastern Colombia, and Venezuela (while in dry forests, they inhabit perennial vegetation and rivers) [25,57,61,62]. Morphologically, *B. asper* has a triangular head, eyes with vertical or elliptical pupils, flat scales, and a yellow jaw (“yellow beard”), and its body coloration may vary from light brown to dark brown with gray or white dots or lines (or cream-colored) that can form triangles or an X when joining the column region [25,63]. The feeding of these animals is quite variable (rodents, centipedes, marsupials, fish, birds, and other snakes) [25,57]. During the day, these snakes are found wrapped in tree trunks, and at night, they are agile, agitated, and dangerous hunters; their venom is considered one of the most active and toxic [25,62]. The bite of this species causes local bleeding in the gums and nose, edema, pain, fever, gastrointestinal bleeding, hematuria, hypotension, nausea, and vomiting [63].

The composition of *B. asper* venom varies in different stages of life (young and adult) and according to the geographic region [24]. Caribbean adults have a composition of about 28.8% PLA_2_s, 18.2% SVSPs, 9.2% LAAOs, and 41% SVMPs, while newborns from the same region have 23.7% PLA_2_s, 6.7% SVSPs, 2.5% LAAOs, and 63% SVMPs [25]. On the other hand, Pacific adults have a composition of about 45.1% PLA_2_s, 4.4% SVSPs, 4.6% LAAOs, and 44% SVMPs, and neonates have 27.7% PLA_2_s, 2.6% SVSPs, 3.4% LAAOs and 65.5% SVMPs [25]. Several isolated components have been characterized and studied for their anti-thrombotic potential, such as BaP1 (SVMP-PI), BaH1 (SVMP-PIII), and Basparin-A (SVPM-PIII), described below [64,65].

### 2.5. Cerrophidion 

The *Cerrophidion* genus (montane vipers) inhabits subtropical regions at medium to high altitudes (1200 and 3500 m) in southern Mexico, in the highlands of Central America west of Panama [66]. These snakes are medium in size (50 to 55 cm) and pale yellow around the mandibular area [66]. The primary representative of this genus is *Cerrophidion godmani* [66,67]. Members such as *Cerrophidion wilsoni* and *Cerrophidion sasai* are found in the region. Still, there are no studies of the venom of *C. wilsoni* and just a few studies of the venom of *C. sasai*.

#### *Cerrophidion godmani* 

This viper is distributed across the plateau of southern Mexico to western Panama and Nicaragua in pine forests, cloud forests, low-altitude dry forests, and mountain forests [66,67,68,69]. *C. godmani* is a nocturnal animal that feeds on small mammals (rodents and shrews) but also lizards and other snakes. Arthropods constitute an essential portion of the diet of juveniles; birds and amphibians are occasional prey [67]. Morphologically, the snake is robust with a relatively long head, the cantal crest is distinct and raised with scales of three to seven intersections at the top of the head between the supraoculars, the tail is short and not pre-elastic, and the jaw tusks are relatively short [68]. This species has a color that matches the soil, with shades ranging from brown, grayish brown, or reddish brown to dorsal and lateral dark brown, with smaller patches forming a dorsal zigzag stripe [68,69]. Studies have shown that this animal’s venom contains a high proportion of SVMPs (32.8%) plus PLA_2_s (23.4%), followed by SVSPs (19.1%) and other proteins such as disintegrins and peptides [66].

Due to the amount of PLA_2_s, this venom is characterized by its myotoxic activity [70] and its high similarity to the venom of *Metlapilcoatlus mexicanus*, which share the same leading protein families [66]. Two types of basic PLA_2_s were found in the venom of *C. godmani*: myotoxin I (Asp 49-PLA_2_) and myotoxin II (Lys 49-PLA_2_). Myotoxin I exhibits high phospholipase activity, while myotoxin II acts upon membranes through a Ca^2+^-independent mechanism that does not rely on catalytic activity [70,71,72]. These PLA_2_s have demonstrated myotoxic action in vivo [70] and in vitro [56]. Additionally, the crude venom of *C. godmani* has anti-coagulant properties, as it can inhibit Factor Xa, XIa, and prothrombinase complex formation and exhibit fibrinogenolytic activity [48,66]. Furthermore, the venom of *C. godmani* can bind to α-1 nAChR orthosteric sites, indicating potential postsynaptic neurotoxicity with higher selectivity for non-mammalian targets [48]. Interestingly, the RGD disintegrin sasaimin, isolated from the venom of *C. sasai*, has anti-platelet aggregation and endothelial cell adhesion properties [73], indicating that the venom of *C. godmani* may also contain promising disintegrins.

### 2.6. Crotalus

*Crotalus* is a genus only found in America, from Southern Canada (in vegetation regions such as pine forests) down to Argentina, whose characteristic is the presence of a rattle at the tail end [74]. The prominent representatives of the Central American members of this group are the species *Crotalus simus* and *Crotalus tzabcan* [74,75].

#### 2.6.1. *Crotalus simus*

*Crotalus simus*, also known as a rattlesnake, is a robust snake (can reach 180 cm in length but usually ranges from 130 to 160 cm) of terrestrial habits, distributed in semi-arid regions with dry to arid tropical climates (absent in rainforests) [3]. This viper is found in Mexico, central west Costa Rica, western Honduras, and Guatemala [3]. Regarding its morphology, the species has a large triangular head, eyes with vertical or elliptical pupils, scalded scales, and a segmented tail-like rattle. A *C. simus* bite causes local effects, with pain, swelling, blisters, and necrosis, which can lead to fasciotomies (surgical opening of compartments to relieve internal pressure) and even amputation [76]. Systemic disorders range from mild to moderate, with hemostatic imbalances, spontaneous bleeding (rare), and neurotoxicity [76].

The composition of the venom of the leading representative member, *C. simus*, has been characterized biologically and biochemically. The venom of adult members is composed of 22% PLAs, including crotoxin, 30% of SVSPs, 28% of SVMPs, 17% of less abundant proteins, such as LAAOs, and 3% of non-identified proteins [10]. A disintegrin called simusmin was purified from this venom [73]. Despite reports on the hemorrhagic activity of the venom from *C. simus*, no studies focus on the potential activity of isolated compounds from this venom against thrombosis. However, the venom compounds, such as crotoxin and LAAOs from other members of the *Crotalus* genus, have demonstrated important potential against thrombosis. For example, the LAAO of *Crotalus adamanteus* displayed pro-coagulant and anti-coagulant activity in human plasma [11]. Likewise, the crotoxin of *Crotalus durissus terrificus* demonstrated significant potential against thrombosis, lowering the levels of vWF and t-PA and elevating the levels of protein C and PAI-1 in HUVEC cells [77]. The venom from *C. simus* has anti-coagulant and anti-inflammatory (action of crotoxin) pro-coagulant activity in vitro and is well known for its hemorrhagic activity [10].

#### 2.6.2. *Crotalus tzabcan*

This terrestrial and daytime snake can reach 180 cm TL [78]. Morphologically, it has a robust body, large head, short tail, and segmented horn-shaped rattle [78]. In addition, it has a very evident spinal ridge with keeled and tuberculated dorsal scales. This species is distributed along the Yucatan Peninsula, northern Guatemala, and northern Belize [78], inhabiting thorn forests and tropical forests with elevations up to 700 m in altitude. This snake feeds on lizards, rodents, birds, and small mammals [3,42]. The proteomics of *C. tzabcan* venom is very similar to *C. simus*, but the main difference is in the amount of neurotoxin (crotoxin). While *C. tzabcan* has 3.0–7.7% of this toxin, *C. simus* has a much higher percentage (7.6–44.3%), which explains the higher lethality of the venom of this latter species [78]. The disintegrin tzabcanin was purified from this venom [73].

### 2.7. Lachesis 

*Lachesis* is a genus of the *Viperidae* family found in remote areas in Central and South America. Their members comprise the longest vipers in the world, with adults varying 2–4.5 m in TL. Their venom is characterized as causing local tissue damage, nausea, coagulopathies, hypotension, bradycardia, diarrhea, vomiting, shock, and renal disturbances. The members found in Central America are *L. stenophrys*, in the Caribbean coast of Central America, *L. melanocephala*, with a distribution restricted to a small area of the Corcovado National Park along the Pacific coast in Costa Rica, and *L. acrochorda*, found in Panama [29]. There are only a few compounds isolated from this venom. Studies have not focused on anti-thrombotic effects.

#### 2.7.1. *Lachesis stenhophrys*

*L. stenhophrys* is a member that presents a predominantly unpigmented head. It possesses a few ventral scales, 197–211 in Females and 215–234 in males. Its name comes from the Greek *stenos*, meaning narrow, and *ophrys*, meaning brow, referencing their small supraocular scales. The venom comprises 30.6% of SVMPs, 27.1% of Vaso active peptides (VAPs), 21.2% of SVSPs, 14.1% of PLA_2_s, 3.6% of CTLs, and 2.7% of LAAOs. Two components have been isolated from this snake: a non-hemorrhagic SVMP-PI of about 24 kDa named LSF, able to degrade α and β-chains of fibrinogen [79,80], and a PLA_2_ of about 13.8 kDa, named LsPA-1, which was not thoroughly studied [79].

#### 2.7.2. *Lachesis melanocephala*

*L. melanocephala* is distinguished by having the dorsum of its head completely black. Its name comes from the Greek *melanos*, meaning black, and *kephalos*, meaning head, referring to its head. Its venom comprises 30.2% of VAPs, 21% of SVSPs, 19% of SVMPs, 13.4% of PLA_2_s, 7.5% of CTLs, and 3.6% of LAAOs. Only one coagulant proteinase was purified from this venom, which needs better characterization. The proteinase is about 30 kDa and can degrade only the α chain of fibrinogen [28].

#### 2.7.3. *Lachesis acrochorda*

*L. acrochorda* can be found from Panama to Ecuador. It is distinguished by having a brown head with black postocular stripes (1–2 scales wide), yellow supralabials, a white throat, and a dark brown body with 23–31 irregular or incomplete black dorsal rhombuses separated by white scales. Its TL is 2.32 m for males and 2.34 m for females. Its name comes from the Greek root of the word acrochordon, which means wart, and this references the warty dorsal scalation that this species presents. Its venom mainly comprises 35.1% of SVSPs, 23.2% of SVMPs, 21.2% of VAPs, 7.9% of CTLs, 2.3% of PLA_2_s, 2.7% of LAAOs, and 1.8% of CRISPs [29,80]. Only one compound is characterized by this venom, a PLA_2_ named Lacro_PLA_2_ of about 13.97 kDa, involved in edema and myonecrosis effects from the venom [81].

### 2.8. Metlapilcoatlus 

The genus *Metlapilcoatlus* is called *Náhuatl metlapil*, referring to the thick mortar used with a grinding stone called *metate* and *coatl* for snakes. These vipers are characterized morphologically by their thick, robust, and short-length bodies [19,47]. The head is large, the eyes are relatively small, the snout is round, and the tail is short, representing about 15% of the body length [19,47]. The color of these snakes varies from gray, brown, or reddish, covered with various lateral and dorsal spots, and their distribution includes Mexico and Central America [19,47]. The most representative species of this genus is *M. mexicanus* [19,47]. The name of this genus recently changed from *Atropoides* to *Metlapilcoatlus* [82,83].

Because of the recent changes in the genus name, most of the data available from this genus are based on studies published under the name *Atropoides*. On the other hand, only some studies of the genus *Metlapilcoatlus* have been published. The venom composition of this genus majoritarily comprises SVMPs and SVSPs. Extraordinarily, the venom of *M. mexicanus* contains three-finger toxins (3FTx) [18,20,48,84]. Despite the potential and uniqueness of the venom of this genus, only a few studies have focused on purified venom compounds and their biotechnological potential [18,20,48,84]. 

#### *Metlapilcoatlus mexicanus* 

*M. mexicanus,* also known as stone hand, is found in several habitats, mainly in the rainforest, tropical regions affected by agriculture (they can be seen in coffee and corn crops), rainforests, and subtropical wet and dry forests [3]. This snake is terrestrial, with nocturnal habits, and its diet includes rodents, birds, reptiles, and amphibians [3]. The adult species of this animal can reach 40–60 cm of TL, but some can reach 80 cm. Morphologically, it is characterized by having a large triangular head, eyes with a vertical pupil, a thick, short body with scales, and a solenoglyph prosthesis, typical of the *Viperidae* family [53]. As for staining, *M. mexicanus* has a dark brown head with or without dark parts, and a dark line can be found from the eye to the mouth, where approximately 15 to 20 brown diamonds are extended throughout the body. Guatemalan species usually have a dark brown diamond with a purple part [3]. This snake has aggressive behavior and, when threatened, attacks by jumping off the ground and biting uncontrollably; due to their color, they can easily merge with the forest floor. The venom of this viper is not very dangerous, which makes bite symptoms mild, with mild swelling, pain, and bleeding in the injured area; however, it can become dangerous because it is a large, robust animal that often traps the limb it attacks. The main toxins of *M. mexicanus* belong to the families of PLA_2_s (relative abundance, 36.5%) and SVSPs (22%) [20]. Most studies have used crude venom, and no proteins have been purified.

### 2.9. Porthidium

*Porthidium* is a genus of small species (adults 55–75 cm long) with terrestrial habits [3], found in low- and medium-altitude forests in Central and South America [85,86,87]. The main characteristic of the members of this genus is the upward slanting of the muzzle [87].

Studies that have focused on the venom of the members of *Porthidium* are scarce. Still, clinical reports indicate that the venom of *P. nasutum* and *P. ophryomegas* may have an anti-coagulant effect on human plasma [84]. Also, members of this genus in South America have shown pro-coagulant activating upon Factor XII of the coagulation cascade to form firm blood clots in vitro. *P. lansvergii*, from Colombia, has disintegrins as venom components; these molecules are known to interfere with platelet aggregation [88].

#### 2.9.1. *Porthidium nasutum*

*P. nasutum*, also known as Bocourt or *tamaga*, is a small animal (females reach a maximum of 65 cm, and males are even smaller) [3], moderately robust, inhabiting tropical forests and lowlands in the Atlantic side of Central America, Ecuador, Venezuela, Colombia, Panama, and southern Chiapas, Mexico [87]. This species has a triangular head with an upward-facing rostral scale (typical of this group) [87]. Its body color may vary from brown to reddish brown, yellowish brown, grayish brown, and gray. Most have a narrow reddish vertebral line and a series of 13–23 dark quadrangular points along each side, with 21–25 (usually 23) lines in the dorsal region on the body scale [87]. The belly is pale to gray, with darker patches [87]. This viper is active day and night (feeding on lizards, frogs, small rodents, and other snakes). It is often seen sunning on shrubs or small trees [87]. 

The venom of this animal can be toxic (hemotoxic with necrotic tissue factors), causing local pain and swelling [86]. An analysis of *P. nasutum* venom revealed a significant percentage of SVMPs (52.1%), followed by PLA_2_s (11.6%), CTLs (10.4%), disintegrins (9.9%), and SVSPs (9.6%) [57]. Only a few reports show the action of purified components, one PLA_2_ and nasulysin-1, an SVMP [31,89].

#### 2.9.2. *Porthidium ophryomegas*

*P. ophryomegas* is distributed throughout Costa Rica, El Salvador, Guatemala, Honduras, and Nicaragua, inhabiting dry areas with thorn bushes and dry lowland forests [3,66]. This snake is the largest within this genus (reaching a maximum of 80 cm). Its diet mainly includes lizards, frogs, and small mammals [3,66]. The body of this animal may be brown, gray, or grayish brown, with a narrow white, yellow, or rust-brown intermediate line, with 23–28 (usually 25) dorsal scale lines of the body and 24–40 dark diamonds along each side [3,66]. Its belly is paler with heavy dark brown spots (especially along the front edge of each ventral scale); its tail is strongly spotted at the basal half, closest to the tip, and its muzzle faces upwards [3,66]. 

The venom composition of *P. ophryomegas* is similar to *P. nasutum*, differing in the percentage of its constituents: SVMPs (45.0%), followed by PLA_2_s (13.5%), CTLs (8.0%), disintegrins (16.7%), SVSPs (7.3%) [31], and the presence of a phospholipase A_2_ pentameric, called PophPLA_2_, with mild anti-coagulant activity [90].

## 3. Potential Biotechnological Applications of Venom Components from *Viperidae* for Thrombosis

### 3.1. Thrombosis 

Thrombosis is a complex pathological process involving blood clots, known as thrombi, within the vascular system. At the biochemical level, thrombosis encompasses various molecular and cellular events. It is intricately linked to the hemostatic system, a series of tightly regulated cellular and biochemical reactions that form a stable blood clot. The coagulation cascade involves activating clotting factors, such as fibrinogen, which is converted to fibrin, a mesh-like protein that forms the structural basis of the blood clot [91]. Various clotting factors like Factor VIII, IX, and X participate in coagulation.

Platelets also play a crucial role in thrombosis, adhering to exposed collagen and becoming activated upon blood vessel injury. This activation triggers a cascade of events, including releasing chemicals, such as thromboxane A_2_, ADP, and serotonin, that further activate additional platelets and enhance coagulation. Endothelial dysfunction can also trigger the formation of thrombi since the endothelium regulates the balance of pro- and anti-coagulant factors in the blood. While many factors can affect this balance, one of the most prevalent is inflammatory conditions that lead to injuries in the inner lining of blood vessels [91,92,93], producing a pro-thrombotic state. Inflammatory molecules, such as cytokines, can activate platelets and endothelial cells, promoting a pro-thrombotic environment.

Anti-coagulant mechanisms, including proteins like anti-thrombin and protein C, counterbalance these pro-coagulant factors to prevent excessive clot formation [94]. Snake venoms that act with an anti-thrombotic activity are summarized in Table 1. Fibrinolysis is the process by which blood clots are broken down. Plasmin, an enzyme, is key for degrading fibrin into soluble fragments. An imbalance between coagulation and fibrinolysis can contribute to thrombotic disorders [95].

**Figure 2:**
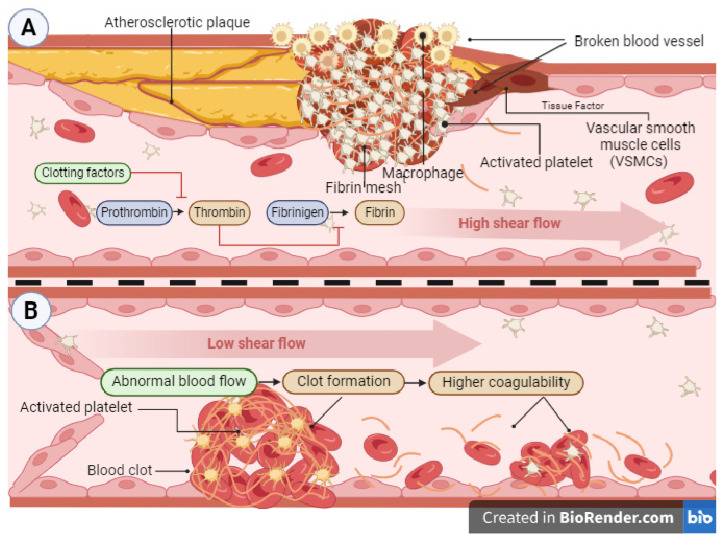
Main types of thrombosis. Thrombosis can be divided into two categories: (**A**) arterial thrombosis, being characterized by the presence of an injury in the blood vessel provoked by an atherosclerotic plaque that activates the production of factors that result in the activation of platelets and the coagulation cascade and (**B**) venous thrombosis, which is characterized by abnormal blood flow conditions inside the vessel that favor the formations of a blood clot; these conditions tend to be multifactorial and include a range of underlying conditions and external stress that can predispose a patient to the production of clots.

Thrombosis, the designated name for a group of coagulopathies, can also be divided depending on the type of blood vessel the thrombi is on [96]. Arterial thrombosis (Figure 2A) is commonly associated with atherosclerosis, the atherothrombotic process. The process of arterial thrombosis is initiated by atherosclerotic plaque rupture, exposing cholesterol, collagen, necrotic tissue, foam cells, and tissue factor in the endothelium. This leads to platelet adhesion and aggregation on the injured artery wall, forming a thrombus. This buildup reduces blood flow as more platelets are added to the thrombus [97,98], leading to partial or total blockage of blood flow. Activated platelets and tissue factor exposition in the plaque and smooth muscle cells activate the coagulation cascade [96,97,98,99]. A venous thrombosis (Figure 2B) can occur in the form of deep vein thrombosis (DVT) or venous thromboembolism (VTE). Changes in blood flow, vascular injury, and hypercoagulability are causative factors in forming a blood clot in the vein. These elements trigger platelet activation and expose the tissue factor, resulting in thrombus formation [100].

Arterial clots are rich in platelets and are formed due to high shear stress, whereas venous clots are rich in fibrin and are formed under low shear stress without requiring any damage to the endothelium. Under normal conditions, the internal surface of the vein is usually covered with anti-coagulant factors that prevent the formation of aggregations. However, several factors can alter this balance and produce conditions that allow for coagulation. The most common causes are abnormal flow or total stasis, alterations in the blood composition that favor coagulation, and unrelated alterations in the endothelium [101,102].

### 3.2. Venom Components

Venoms are secretions released by venomous animals such as snakes and lizards and are formed by complex mixtures of organic and inorganic compounds of great pharmacological and therapeutic value [103]. Venoms are stored and synthesized in specialized glands and are released for defense, immobilization, and prey capture [103,104]. The protein composition of these venoms can vary qualitatively and quantitatively between species depending on region, climate, and different ontogenetic stages (developmental stages from birth to adulthood) [103,105]. Venomous secretions can produce harmful effects and even death of victims affected by bites [104,106]. However, the molecules in these venoms have interesting characteristics, such as specificity and selectivity to ion channels, receptors, and enzymes, as well as catalytic efficiency, thermal stability, and resistance to proteolysis [106]. This fact has attracted researchers’ attention to the study of the proteomics of these venoms and the isolation of their constituents for application in medical therapy and biotechnology [104,106].

**Figure 3:**
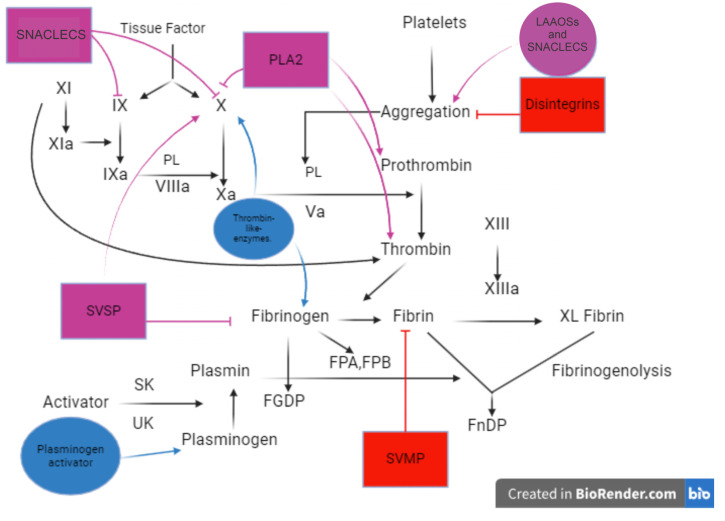
Coagulation cascade and snake venom protein families with anti-coagulant (red), pro-coagulant (blue), and both activities (purple). The abbreviations mean the following components: Snaclecs (snake C-type lectin-like proteins), Phospholipids (PLs), Fibrinopeptide A (FPA), Fibrinopeptide B (FPB), Fibrinogen degradation products (FGDPs), Fibrin degradation products (FnDPs), Urokinase (UK), Streptokinase (SK) and Cross-Linked fibrin (XL Fibrin).

A proteomic analysis of both *Viperidae* snake venoms indicates that their principal constituents are zinc-dependent metalloproteinases (SVMPs), phospholipases A_2_ (PLA_2_s), serine proteinases (SVSPs), disintegrins, and peptides, followed by other protein types present in various proportions, depending on the venom, such as L-amino-acid oxidases (LAAOs), hyaluronidases, C-type lectin proteins (CTLs), and cysteine-rich secretory proteins (CRISPs), among others [107,108]. The main classes of snake (of the family *Viperidae*) venom components and some of their isolated components with possible anti-thrombotic activity are described in Table 2 and Figure 3.

Understanding the relationship between the interaction of isolated components of venom and physiological mechanisms allows for the application of these proteins or their derivatives for specific purposes. Thrombosis is one of the most prevalent conditions in the world. It is of particular interest to study the mechanisms that intervene in aspects of thrombosis and could have anti-thrombotic potential [127]. This review will focus on some of these components purified from *Viperidae* venoms worldwide, including, in Central America, venoms that could have similar components but still need to be explored.

Captopril is an important anti-hypertensive drug developed based on the BPP isolated from the venom of *Bothrops jararaca*. It is the first natural inhibitor of angiotensin-converting enzyme I (ACE) [128,129]. Some toxins from viper venom have been used in thrombosis due to their potential as models for new drugs and diagnostic methods [130]. Tirofiban is an anti-platelet agent indicated for patients with coronary ischemic syndrome [131]. Its structure is derived from echistatin, an RGD disintegrin present in the venom of *Echis carinatus*, which recognizes the platelet αIIBβ3 receptor [132]. Eptifibatide is another platelet anti-aggregating agent originating from a disintegrin: barbourin, a KGD disintegrin found in the venom of *Sistrurus miliarius* [133,134].Tirofiban and eptifibatide can lead to bleeding and thrombocytopenia [131], which is why the search for safer drugs is continuing. Batroxobin, also known as reptilase, is a thrombin-like enzyme isolated from the venom of *Bothrops atrox*. Reptilase cleaves fibrinogen, releasing fibrin, and is used in the laboratory clotting assay RT (reptilase time) [135,136,137]. Protec is a protein C activator (APC) derived from the snake *Agkistrodon contortrix*, which, in addition to having potent anti-coagulant action, is utilized as a tool in the laboratory diagnosis of disorders in the protein C pathway [138,139].

Notably, most drugs derived from animal toxins approved for medical and laboratory use participate in hemostatic processes. However, despite their significant biotechnological potential, many venoms with different compositions and proteins featuring different interaction pathways still need to be explored. Central America is part of this research interest, as it has microclimates in geographically close regions, resulting in selective pressures that generate a diversity of niches occupied by different genera and species of venomous animals. Therefore, the families of proteins of interest obtained from *Viperidae* snake venoms that correlate with any of the pathways that develop the formation of a thrombus are described as the leading interest for potentially treating this condition [140].

#### 3.2.1. Metalloproteases and Disintegrins

Proteases are the most abundant in snake venom, differing in two broad classes according to their structure: metalloproteinases and serine proteases [109]. Metalloproteinases (metalloproteinases and disintegrins) belong to the family of metzincins and matrix metalloproteinases and are characterized by a zinc-dependent domain in their structure [108,111,112]. These proteinases are distributed into three groups: P-I, P-II, and P-III [108,111,112]. 

P-I is the simplest class, with only one metalloproteinase domain, with fibrinolytic, low hemorrhagic, myonecrotic, proinflammatory, and apoptosis-inducing functions [108,111,112]. P-II contains a disintegrin and a catalytic domain. The disintegrin is released after the cleavage between domains, probably during venom formation. The disintegrin hosts a canonical RGD (arginine–glycine–aspartic acid) sequence [112,113]. Disintegrins can be used in cancer therapy as anti-angiogenic (angiogenesis is the growth of new blood vessels), anti-metastatic (metastasis is the invasion of cancer cells to other organs or tissues), anti-proliferative, and apoptosis inducers, as they selectively block the integrins (adhesion receptors) involved in the development of these pathologies [141]. P-III is formed by a catalytic, followed by a disintegrin and cysteine-rich domains [108,112]. P-III has pro-inflammatory and apoptosis-inducing hemorrhagic action, acting on basement membrane proteins and endothelial cells and inhibiting platelet aggregation (collagen-dependent) [108,112]. Some P-III may have an additional lectin domain and activate clotting factors [108,112]. 

Many metalloproteinases and disintegrins have been characterized. Below, we present some of these molecules from *Viperidae* venoms from the American continent characterized, showing their potential as tools for developing anti-thrombotic drugs.

##### Jararhagin

Jararhagin is a P-III metalloproteinase of 52 KDa molecular weight isolated from *Bothrops jararaca* venom. It belongs to the subfamily of soluble zinc-dependent metalloproteinases, and its structure consists of a catalytic domain, followed by an ECD-disintegrin-like (ECD = Glu-Cys-Asp) domain and a Cys-rich adhesive domain [142]. Even though this enzyme does not have a crystallographic structure available, its isoform called Bothropasin does. This homolog structure shares 95% of its identity with jararhagin (with only 19 different residues, mainly distributed in the catalytic domain), and it has yet to be as well studied as jararhagin. Still, their high level of similarity could indicate that these enzymes share similar biological properties [143].

This toxin exhibits anti-tumorigenic properties demonstrated by experimental studies [109,110]. In vitro (mouse and human SKmel-28 cells) and in vivo (rat AIRmin) studies showed that jararhagin could inhibit pulmonary melanoma adhesion, migration, invasion, and metastasis induction [109]. It also promoted endothelial cell-specific apoptosis, with the activation of pro-caspase 3 (a protease capable of activating apoptosis) and alteration of the Bax/Bcl pathway (Bcl encodes proteins associated with cell membrane protection against apoptosis, and Bax is a Bcl antagonist that promotes cell death). Apoptosis was further followed by decreased cell viability, loss of adhesion, and induction of changes in cell format [109]. Jararhagin was also used for in vivo treatment of murine melanoma cells (B16F10), where the result showed a reduction in the incidence of nodules and metastases, with increased apoptosis through activation of the caspase3 pathway and decreased cell viability [110].

Moreover, one of the most studied actions of jararhagin is related to the inhibition of platelet aggregation through two pathways that require the involvement of ristocetin and collagen. In the case of the ristocetin pathway, the mechanism of action has been associated with the direct hydrolysis of a collagen-bound factor, von Willebrand factor (vWF), by the toxin, preventing platelet activation, and it has not been associated with an interaction with the receptor GP Ib-IX-V, the mediator of platelet adhesion [144]. In the case of the collagen-dependent option, the inhibition seems to occur when the toxin interacts with the integrin receptor α_2_β_1_, showing no effect on the von Willebrand factor (vWF) or a collagen receptor called GPVI, equally deactivating the platelet response to stop bleeding [144,145]. Both pathways result in valuable approaches for considering this toxin as an appropriate tool for developing anti-platelet drugs.

##### Crotalin

Crotalin, a potent platelet glycoprotein Ib (GPIb) antagonist, is an SVMP-PI of ~30 kDa, purified from the venom of *Crotalus atrox*. This metalloproteinase exhibits an anti-thrombotic effect when intravenously administrated to mice; the mechanism of action demonstrated that this enzyme could inhibit vWF binding and glycoprotein Ib (GPIb) activity. Despite the potential and uniqueness of this SVMP, there are only a few studies of its mechanism and uses as an antithrombotic agent [146,147].

##### *B. asper* Venom Metall Oproteinases That Interfere with Blood Compounds

Four metalloproteinases from *B. asper* venom have been identified: BaH1, BH4, Basparin A, and BaP1. The characterization and local hemorrhage effect of BaP1 is well known. BaP1 is a P-I metalloproteinase of 24 kDa that presents hemorrhagic effects. This metalloproteinase can degrade casein and fibrinogen, with a rapid degradation of the α-chain and a slower degradation of the β-chain of human fibrinogen solution [148]. Also, BaP1 has hemorrhagic activity in intradermal and intramuscular injections in mice [148]. BaH4 is an acidic hemorrhagic P-III metalloproteinase of 64 kDa, with a lower activity over fibronectin and high induced hemorrhagic activity after intradermal injection in mice [149]; Basparin A is a 70 kDa P-III metalloproteinase that inhibits collagen-dependent platelet aggregation in vitro, without inducing hemorrhage, myonecrosis, or edema in mice [150]. Finally, BaH1 is a P-III hemorrhagic metalloproteinase that is mainly responsible for the hemorrhagic activity of *B. asper* venom [65,150]. Despite these effects, no study has focused on the potential anti-thrombotic effects of these metalloproteinases.

##### Tzabcanin

Tzabcanin is a disintegrin (P-II) isolated from the venom of the snake species *Crotalus tzabcan*, which contains an RGD (Arg-Gly-Asp tripeptide binding) domain and has a molecular weight of 7.1 kDa [7,8]. In vitro studies have shown that this compound has anticancer activity by inhibiting cell adhesion and the migration of melanoma (A-375), lung cancer (A-549), colorectal adenocarcinoma (Colo-205), and breast adenocarcinoma (MCF-7) [7,8]. These cancer cells express αvβ3, an integrin receptor for various extracellular matrix proteins (ECM), such as vitronectin and fibronectin (the binding between αvβ3/ECM through the ECM RGD domain) [7,8]. Increased expression of this integrin stimulates phosphorylation of the Map/Ras protein kinase pathway (mitogen-activated protein pathway/signaling molecule) in the production of specific genes for cell growth, differentiation, and migration, facilitating angiogenesis and metastasis [7,8]. Tzabcanin, through its RGD domain, binds to αvβ3, inhibiting β3 subunit/vitronectin binding and, consequently, adhesion and migration of new cancer cells [7,8]. No investigation on the anti-thrombotic effect was performed for this disintegrin.

##### Jararacin/Jarastatin

Jararacin and Jarastatin are middle-class disintegrins found in *B. jararaca* venom. Both weigh 7.7 kDa, and both induce the inhibition of platelet aggregation induced by ADP, thrombin, and collagen in human platelets in vitro. Jararacin was first isolated by Scarborough in 1993 and possesses a high affinity for the integrin αIIbβ3 and a lower affinity for αvβ3 [151,152,153,154,155,156,157]. On the other hand, jarastatin was first discovered by Zingali et al. (2009) and was demonstrated to have a higher affinity for the integrin αvβ3 and a lower affinity for the integrin αIIbβ3 [151,152,153,154,155,156,157]. Both disintegrins show properties as anti-thrombotic agents; in the case of jararacin, it diminishes thrombus formation in vivo without causing bleeding. Other than this, both are under structural studies to understand the mechanisms that can guide researchers in creating an anti-thrombotic agent. Like these disintegrins, these molecules from Viperidae venoms from Central America have the same potential as new anti-thrombotic agents.

#### 3.2.2. Phospholipases A_2_ (PLA_2_s)

PLA_2_s (EC 3.1.1.4) are low molecular weight enzymes (ranging from 13 to 19 kDa) that catalyze the hydrolysis reactions of sn-2-acyl binding of sn-3-phospholipids, resulting in free fatty acid and lysophospholipid products [90]. These enzymes have a variety of pharmacological effects such as anti-coagulant, hemolytic, neurotoxic, myotoxic, edema inducer, hemorrhagic, cytolytic, cardiotoxic, muscarinic inhibitor, platelet anti-aggregant, and anti-coagulant effects [90]. Recent studies have revealed that PLA_2_s have anti-inflammatory, anti-tumor, and anti-angiogenic activity [80,90,158]. Also, snake venoms have some PLA_2_-like toxins, but there is no evidence of an anti-thrombotic activity of these enzymes, and we will not discuss them [158,159,160].

Phospholipase A_2_ enzymes present variability in the intensity of anti-coagulant activity, although only the mechanism of action of some of these proteins is known. It is important to emphasize how, within the same family of proteins, it is possible to attack several steps of the coagulation cascade and to implement treatments that better adapt to the patient’s condition [161]. PLA_2_s, with the highest anti-coagulant activity, block the activation of Factors X and Xa through the extrinsic tenase complex [162]. Likewise, other phospholipases with intense anti-coagulant activity inhibit prothrombin activation by binding to Factor Xa, thereby interfering with the prothrombinase complex [163]. Due to the high variation between activities, much is still unknown about how other PLA_2_s may vary in interaction and concentration at the tissue level [163]. Moreover, a PLA_2_ isolated from *P. nasutum* venom exhibited anti-bacterial activity against Gram-positive bacteria [89], whereas another found in the venom of *P. ophyromegas* showed the myotoxic, cytotoxic, and anti-coagulant effects mentioned above [90].

Below, we show other possibilities for studies with a well-known toxic PLA_2_ molecule, crotoxin.

##### Crotoxin

Crotoxin is found in the venom of the *Crotalus* snake genus (*C. durissus*, *C. simus*, and *C. tzabcan*). It is a β-heterodimeric neurotoxin formed by the non-covalent association of two units: a basic subunit (PLA_2_) and an acidic non-enzymatic subunit (crotapotin) with a molecular weight of 24 to 26 KDa, an isoelectric point of 4.7. According to crystallographic analysis, the molecular ratio between these subunits is 1:1 [164]. It was the first snake venom protein to be crystallized, and it is known to have neurotoxic activity as a post and presynaptic blocker. It has also been described to have anti-inflammatory, antitumor properties and anti-angiogenic activity by inhibiting the pro-angiogenic factors TNF- α (tumor necrosis factor-α), VEGF (vascular endothelial growth factor), and matrix metalloproteinases (MMP-2 and MMP-9), also inhibiting the anti-angiogenic factors LXA4 (lipoxin A4) and its stable analogs (15-epi-LXA4) [4,5]. There are more activities of crotoxin, but we will focus on those that affect thrombosis [4,5,10,77,164].

The anti-inflammatory activity of crotoxin was evaluated by several techniques in vivo and in vitro [114,115]. Briefly, this toxin decreased paw edema, and carrageenan-induced polymorphonuclear migration promoted decreased cell adhesion on the surface of the cremaster endothelium microcirculation; significantly decreased the levels of inflammatory cytokines TNF-α (tumor necrosis factor-α), IL-1β (interleukin-1β) and IL-6 (interleukin-6) in the injured tissue; and increased IL-10 anti-inflammatory mediators (interleukin-10), TGF-β (transforming growth factor β), LXA4, and PGE2 (prostaglandins 2) at the injured site [114,115,165].

**Figure 4:**
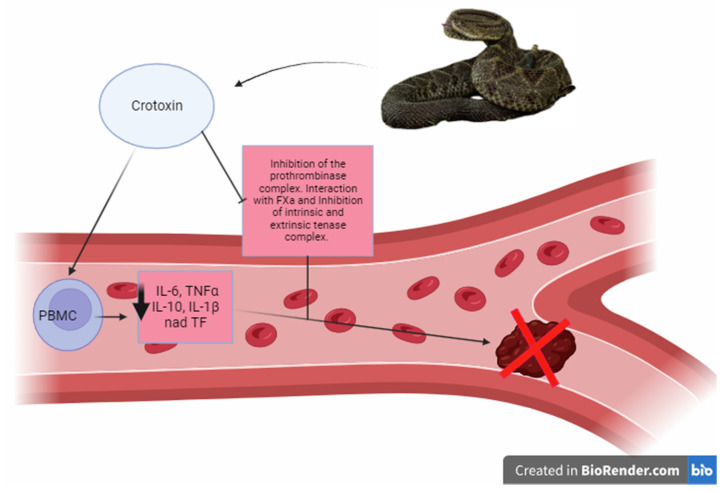
Role of crotoxin as a coagulation modulator. The figure shows the inhibition of the prothrombinase complex and the intrinsic and extrinsic tenase complex by crotoxin, as well as the diminution of inflammatory cytokines IL-6, TNF-α, and IL-1β that may contribute to a thrombus inhibition.

There is crosstalk between the anti-inflammatory effect of crotoxin and coagulation modulation: peripheral blood mononuclear cells (PBM) stimulated with LPSs (lipopolysaccharides) and treated with crotoxin exhibit a decrease in the production of pro-inflammatory cytokines IL-6, TNF-α, and IL-1β. At the same time, tissue factor (TF) expression is reduced, resulting in diminished coagulation activity of these cells in a whole-blood culture [166]. In addition to the anti-inflammatory effect, crotoxin demonstrates anti-coagulant activity in PT (prothrombin time) and aPTT (activated partial thromboplastin time) tests [166,167,168] (Figure 4). The anti-coagulant activity is associated with inhibiting the prothrombinase complex, mediated through the interaction of crotoxin with Factor Xa, replacing Factor Va in the prothrombinase complex [168,169]. Interestingly, crotoxin does not alter the activity of Factor Xa alone [166]. Apart from the prothrombinase complex, crotoxin can also inhibit intrinsic and extrinsic tenase complexes [166]. Crotoxin has also been found to positively affect the endothelium in an in vitro study that used human umbilical vein endothelial cells (HUVECs) stimulated with LPSs. In these cells, crotoxin reduces the levels of vWF and prevents the decrease in tissue plasminogen activator (t-PA) levels. At the same time, it elevates protein C and plasminogen activator inhibitor-1 (PAI-1) levels, which counteracts the pro-coagulant and anti-fibrinolytic effects of endotoxin. It is worth noting that the concentrations of crotoxin used in the study did not have any toxic effects on the cells or interfered with the hemostatic system of cells not stimulated by LPSs. These findings suggest that crotoxin can be a potential therapeutic tool in coagulopathies associated with inflammatory diseases [77].

#### 3.2.3. Serine Proteases

SVSPs are protease components of the peptidase family. They have a characteristic trypsin fold, featuring a highly reactive serine residue within their active site. These enzymes catalyze the cleavage of specific covalent bonds in peptides and proteins. Some of these may affect the blood coagulation cascade, activating coagulation components, fibrinolysis, platelet aggregation, and proteolytic degradation [111,116]. SVSP activities disrupting hemostasis can manifest as pro-coagulant or anti-coagulant [170]. Pro-coagulant proteases are further categorized into those capable of activating Factors II, VII, and/or X, as well as thrombin-like enzymes (TLEs) that cleave fibrinogen into fibrin [171]. Other hemostatic activities involve direct fibrin(ogen)olysis, protein C and plasminogen activation, anti-thrombin, PAI-1, and α_2_-anti-plasmin inhibition. Other SVSPs have kininogen activity similar to kallikrein, releasing bradykinin, a hypotensive peptide [111,116]. Finally, some have unique serine protease activities, such as the activation of Factor V [111,116].

SVSPs with the ability to activate protein C have been shown to have a direct anti-coagulant effect [139]. The importance of protein C comes from the fact that, when circulating in the blood, it is activated by thrombin. Once activated, it can degrade Factors Va and VIIIa of the coagulation cascade, blocking amplification [139]. Another point related to hemostasis is the release of a tissue-type plasminogen activator that stimulates fibrinolysis through interaction with PAI [172]. This activator has been found and studied with greater intensity in the *Agkistrodon* genus; Central America has *A. bilineatus.*

Serine proteases designated as TLEs are capable of depleting fibrinogen within the plasma. Therefore, they do not allow for blood clotting. This effect has yet to be studied sufficiently to determine its clinical potential in treating thrombosis or its direct or indirect impact on thrombi already established in veins or arteries [173]. However, some TLEs act on Factor XIII of the coagulation cascade, degrading the factor itself, which is another exciting approach for searching for anti-thrombotic enzymes in venom. The activation of plasminogen is attractive because it can lead to the destruction of the thrombus by activating fibrinolysis [173]. Below, we present some of the most studied SVSPs from *Viperidae* snakes with promising effects.

##### Batroxobin

Batroxobin is a thrombin-like serine protease from the venom of *B. atrox moojeni* that clots fibrinogen. In contrast to other thrombin-like proteins found in snake venom, Batroxobin only cleavages the α-chain of fibrinogen; also, it is not inhibited by anti-thrombin or heparin cofactor II. In addition, Batroxobin has been used clinically to prevent and treat thrombosis because it can lower circulating fibrinogen levels [9,174,175]. Despite this serine protease belonging to a snake venom from South America, it shows the potential that the venoms of Central America have similar enzymes due to conservations between the *Bothrops* venoms.

##### Plasminogen Activators

Plasminogen activators (PAs) are serine proteases that catalyze the conversion of zymogen plasminogen into the plasmin via proteolytic cleavage of a single peptide bond, enabling fibrinolysis [176]. The first plasminogen activator isolated from snake venom was TSV-PA, found in the venom of *Trimeresurus stejnegeri*. TSV-PA is a single-chain glycoprotein with a molecular weight of 33 kDa, and plasminogen activation occurs through the cleavage of the peptide bond Arg561-Va1562 [177]. Furthermore, it possesses a prolonged half-life due to its structure, allowing it to evade plasma serine protease inhibitors (Serpins) [177]. Other plasminogen activators include LV-PA, derived from *Lachesis muta muta* venom, with a molecular weight of 33 kDa [178,179] and Haly-PA, from *Agkistrodon halys* venom [179]. Another example of a PA is ABUSV-PA, from *Agkistrodon blomhoffii ussurensis* venom, with a molecular weight of 27 kDa and arginine ester hydrolysis activity [180]. The potential anti-thrombotic properties of plasminogen activators have yet to be extensively studied, making it an intriguing area for future research.

#### 3.2.4. L-Amino-Acid Oxidases

L-amino-acid oxidases (LAAOs) are homodimeric flavoenzymes that facilitate the stereospecific oxidative deamination of L-amino-acids, yielding α-keto acids and generating hydrogen peroxide [120]. These enzymes possess a molecular mass ranging from 110 to 159 kDa and consist of three primary domains: a substrate-binding domain, an FAD-binding domain, and a helical domain [119]. The impact of LAAOs on platelet aggregation is contingent upon the specific isoform, with the ability to either inhibit or induce this process. Additionally, LAAOs may be implicated in various physiological responses, including edema, inflammation, apoptosis, hemolysis, and bleeding [119]. It is also recognized that these proteins have a direct interaction with platelet function. This interaction with platelets and possible relationship in hemostasis processes has generated interest in studying the mechanisms of this type of protein in greater depth [118]. In Sakurai et al. (2003), an L-amino oxidase isolated from the venom of *Agkistrodon halys blomhoffii* presented anti-coagulant activity [117]. The most stimulating factor in this type of protein is that, besides affecting Factor IX, this enzymatic effect is due to an intrinsic route and seems to not produce or interact with hydrogen peroxide. However, few LAAOs were studied for their relation to blood clotting [117]. Since venoms of Central American snakes have shown a significant amount of LAAOs, these molecules are attractive targets to be explored.

#### 3.2.5. Snaclecs (Snake C-Type Lectin-Like Proteins)

The C-type lectins (CTLs) from snake venoms are classified into two categories: classical, which have a carbohydrate recognition domain that binds to sugars, and non-sugar-binding C-type lectin-related proteins, known as C-type lectin-like or snaclec (snake C-type lectin-like) proteins. Snaclecs are more prevalent in snake venoms than classical CTLs [181]. While classical CTLs exist as disulfide-linked homodimers or homo-oligomers, snaclecs form disulfide-bonded heterodimers or oligomeric complexes of heterodimers, comprising two closely related subunits tightly associated by loop-swapping [182]. The concave surface between these subunits is likely the primary ligand binding site. Classical CTLs, predominantly binding to galactose, induce platelet aggregation. By contrast, snaclecs do not bind to sugars. They can act as agonists or antagonists of platelet aggregation or exhibit anti-coagulant properties by binding to Factor IX, X, or α-thrombin [121,122,181].

These snaclecs can be classified into three main groups based on their functions: anti-coagulant proteins, platelet aggregation antagonists, and platelet aggregation agonists [121,122,181]. An example of a platelet aggregation agonist is Aspercetin, isolated from the venom of *B. asper*, which induces platelet agglutination by promoting the interaction between vWF and the platelet receptor GPIb [64]. Platelet aggregation antagonists can act in different ways. For example, EMS16 from *Echis multisquamatus* is a selective antagonist of α2β1, the collagen-binding integrin on platelets [183]. Another example is flavocetin-A from *Protobothrops flavoviridis*, commonly known as the habu snake, which binds to the GP Ibα-subunit, inhibiting vWF-dependent platelet aggregation and also targets α2β1 integrin [121]. As representatives of anti-coagulant snaclecs, some proteins bind to coagulation factors, such as IX/X-binding proteins [184,185], IX-binding proteins [123], or bothrojaracin from *Bothrops jararaca*, which acts as a thrombin inhibitor [124]. The number of characterized snaclecs is growing. Below, we present some of these proteins from *Viperidae* venom. It is worth noting that many venoms from Central America have been shown to possess a large amount of these molecules, such as *Bothriechis* and *Porthidium* genera. Below, we show some snaclecs that act on blood factors and platelet receptors as anti-thrombotic molecules.

##### Bothrojaracin

Bothrojaracin is a snaclec with a molecular weight of 27 kDa, isolated from the venom of *Bothrops jararaca* [124]. This protein acts as an anti-coagulant by strongly inhibiting thrombin, specifically. This property has been demonstrated in an in vivo model, showing significant anti-thrombotic activity [186]. Bothrojaracin inhibits thrombin by interacting with exosites 1 and 2 of thrombin. This interaction affects the binding of thrombin to fibrinogen, platelet receptors, thrombomodulin, and heparin receptors [186,187,188]. Bothrojaracin also binds with prothrombin, the precursor of thrombin, inhibiting the activity of the prothrombinase complex [189,190].

##### IX/X-bp (Factors IX- and X-Binding Protein)

Habu IX/X-binding protein (Habu IX/X-bp) is a two-chain anti-coagulant snaclec found in the venom of *Protobothrops flavoviridis*. It was the first snake venom protein discovered to bind to a coagulation factor [191,192]. IX/X-bp binds to the Gla (γ-carboxyglutamic acid) domain of coagulation factors IX and X in the presence of Ca^2+^, blocking the formation of the prothrombinase and intrinsic tenase complexes without inhibiting the enzyme’s active site [193,194]. Another snaclec isolated from the venom of *Protobothrops flavoviridis* is a Factor IX-binding protein, which shares significant homology with IX/X-bp but differs in amino acid residues located at the C-terminal [123]. Sekia et al. (1993) described the presence of a similar protein in the venom of *B. jararaca* named jararaca IX/X-bp. It is interesting to note that Agkisacutacin, isolated from the venom of *Agkistrodon acutus*, is structurally like IX/X-binding proteins but exhibits fibrinogenolytic activity [191,195].

##### Rhodocetin 

Rhodocetin is a protein found in the venom of *Calloselasma rhodostoma*. This controversial protein has a heterodimeric structure of two subunits (αβ)2. Rhodocetin binds to integrin α2β1, which is the collagen receptor on platelets. This binding inhibits collagen-induced platelet aggregation [196,197,198]. However, if integrin α2β1 is absent, rhodocetin triggers platelet activation and aggregation by inducing clustering of C-type lectin-like receptor 2 (CLEC-2) receptors on the platelet surface [199]. Interestingly, the discovery of the CLEC-2 receptor in platelets resulted from studies with rhodocetin. Since then, this receptor has been investigated as a potential target for developing anti-platelet drugs [200].

##### Glycoprotein Ib-Binding Protein

A platelet glycoprotein Ib-binding protein (GPIb-BP) was isolated from the snake venom of *Bothrops jararaca*. This protein was shown to belong to the snaclec family. It binds to this receptor, inhibiting ristocetin- or botrocetin-induced von Willebrand Factor (vWF) binding without affecting the platelet aggregation induced by ADP or alpha-thrombin. GPIb-BP abolished vWF-dependent shear-induced platelet aggregation at high shear stress but not at low shear stress [201]. Other snaclecs that inhibit the GPIb receptor include echicetin, tokaricetin, CHH-A and B, TSV-GPIb-BP, lebecetin, purpureotin, and agkicetin [202]. It is important to emphasize that other snaclecs bind to platelet receptors inducing aggregation or agglutination, which were not in the scope of this review.

#### 3.2.6. Bioactive Peptides

Bioactive peptides are stable molecules capable of resisting the adverse proteolytic environment in the glands of venomous animals [203]. This stability is formed by disulfide bonds and/or post-translational modifications [203]. These peptides’ actions happen quickly before their prey’s internal mechanisms degrade, neutralize, and excrete them from their organism. Thus, these compounds have meaningful therapeutic value with diversified pharmacological action, high affinity, and selectivity for target receptors [203]. These classes are the least explored in Central American snake venoms.

##### Crotamine

Crotamine is a highly basic and non-enzymatic polypeptidic myotoxin with a molecular weight of 4.8 kDa, and it is one of the major constituents of the *C. durissus terrificus* venom. It is formed by only 42 amino acids and six cysteine amino acids that form three disulfide bridges, making this peptide a compact, stable, and positively charged molecule [125,204]. Its myotoxicity effect is carried out by causing muscle contractions generated by the depolarization of the cells due to an increment in the sodium permeability of the membrane [126]. It has been reported that crotamine interferes with the permeability of cellular membranes of lower eukaryotes and prokaryotes, turning it into a candidate for the development of anti-microbial and anti-fungal compounds [125,126,204,205]. Moreover, this peptide has anti-tumoral activity and is also considered a cell-penetrating agent, potentially transporting drugs inside mammalian cells without needing a specific receptor [125,126,204,205]. 

Furthermore, crotamine exhibits a marked anti-coagulant action and increases clot formation time dose-dependently in the prothrombin time (PT) test [168]. This anti-coagulant action may be attributed to its interactions with negatively charged surface areas in the coagulation cascade factors [125,206].

## 4. Conclusions

Central America is a promising region for producing biomolecules from animal venoms, such as those from snakes and lizards. The geographical location of the countries appears to have favored the development and evolution of venom secretions of the species distributed in this territory. A proteomic analysis of the venom of the *Viperidae* species indicates the presence of exciting components with various biological activities such as anti-tumor, anti-cancer, anti-inflammatory, anti-diabetes, anti-hypertensive, and pro-apoptotic effects, including anti-thrombotic potential. However, little is known about the mechanism of action of these molecules, and, to our knowledge, there are no clinical trials involving venom components from Central American snakes. Although the path is time-consuming to clarify all aspects that comprise the action of a biomolecule in the body, the components of these venoms prove to be a good option for the emergence of new pharmaceutical and biotechnological bases due to their selectivity and specificity. These characteristics are essential for the development of new drugs.

## Figures and Tables

**Table 2:** Potential anti-thrombotic applications of enzymes and peptides of Central America *Viperidae* venom components.

Protein Family	Biotechnological Potential for Thrombosis	Reference
Metalloproteases and disintegrins	Inhibition of clot formation in relation to the consumption of fibrinogen; cleavage of platelet receptors; inhibition of platelet aggregation	[7,31,108,109,110,111,112,113]
Phospholipases A_2_ (PLA_2_s)	Strong anti-coagulant proteins that display inhibition of Factors Xa and X at lower concentrations by an extrinsic tenase complex and activation of prothrombin to thrombin.	[114,115]
	Weak anti-coagulant proteins that exhibit inhibition of Factors Xa and X.	[90,114,115]
Serine proteases	Cleaves fibrinogen plasminogen, Factor Va/VIIIa, and protein C in plasma	[111,116]
L-amino-acid oxidases	Isolation of proteins with anti-coagulant activity on factors of the coagulation cascade	[117,118,119,120]
Snake C-type lectin-like proteins (snaclecs)	Inhibition of clotting factors (thrombin, Factor X, and Factor IX); inhibition of platelet receptors	[58,64,121,122,123,124]
Bioactive peptides	Compounds with high affinity and specificity of receptors associated with different biological activities	[125,126]

## Data Availability

Not applicable.
